# Post-Operative Euglycemic Diabetic Ketoacidosis in a Patient With SGLT-2 Inhibitor Use and Recent Sleeve Gastrectomy

**DOI:** 10.7759/cureus.14297

**Published:** 2021-04-05

**Authors:** Alexander Smith, John Holtrop, Moutamn Sadoun

**Affiliations:** 1 Department of Endocrinology, Wayne State University School of Medicine, Detroit, USA; 2 Department of Surgery, Ascension St. John Hospital, Detroit, USA

**Keywords:** diabetes, bariatric surgery, euglycemic, sglt-2 inhibitor, ketoacidosis

## Abstract

A 51-year-old woman with type 2 diabetes mellitus developed euglycemic diabetic ketoacidosis (euDKA) in the post-operative setting after robotic-assisted sleeve gastrectomy. She developed tachycardia on post-operative day (POD) 1 before developing altered mental status and tachypnea on POD 2. The diagnosis was ultimately made by discovering ketonuria in the setting of anion gap metabolic acidosis despite repeatedly normal blood glucose levels. Pre-operatively, her blood glucose levels were managed with sodium-glucose co-transporter-2 (SGLT-2) inhibitor-containing combination pill, Invokamet®, as well as basal-bolus insulin regimen consisting of aspart (NovoLog®) and glargine-lixisenatide (Soliqua®). SLGT-2 inhibitors have been associated with an increased risk of euDKA, particularly in the context of severe bodily stressors such as surgery. EuDKA is a difficult diagnosis to make because of the lack of characteristic severe hyperglycemia that is typical of DKA. Clinicians should be mindful of euDKA in the post-operative setting of diabetic patients, particularly for those on SGLT-2 inhibitors.

## Introduction

Sodium-glucose co-transporter-2 (SGLT-2) inhibitors, such as canagliflozin, are a new class of anti-hyperglycemic medications that have been shown to significantly improve control of blood glucose levels, weight, and blood pressure [1,2]. Mechanistically, these drugs decrease the resorption of glucose at the proximal tubules within the nephron, thereby lowering blood glucose levels. However, despite their efficacy, they carry an increased risk of urinary tract and genital tract infections, lower limb amputation, and, as in this case, euglycemic diabetic ketoacidosis (euDKA) [3,4].

Despite euDKA comprising 3.2% of all DKA admissions [5], it is a diagnosis that can elude skilled clinicians, as the euglycemia masks the underlying ketoacidosis. EuDKA can occur in both type 1 diabetes mellitus (T1DM) and type 2 diabetes mellitus (T2DM), although a vast majority occur in patients with T1DM and is characterized by the following: blood glucose less than 250 mg/dL, severe metabolic acidosis (arterial pH less than 7.3), and serum bicarbonate less than 18 mEq/L [6]. Other risk factors include intense bodily stressors such as recent surgery, major illness, severe food restriction, and a relative insulin deficiency [6].

Although the precise mechanism of how euDKA occurs in the setting of SGLT-2 inhibitor use is unknown, decreased insulin secretion appears to be critical for developing euDKA [7-9]. SGLT-2 inhibitors promote glucosuria, and the resulting decrease in blood glucose levels leads to decreased insulin secretion. Additionally, either through the decreased insulin production or direct stimulation of pancreatic alpha cells, there is an increase in glucagon secretion [10]. This decrease in insulin and increase in glucagon promotes the formation of the inactive, phosphorylated state of acetyl-CoA carboxylase - the enzyme responsible for the committed step in fatty acid synthesis [11]. As a result, SGLT-2 inhibitors shift the body’s metabolic priority away from glucose and toward fatty acids through lipolysis. This results in an increase in free fatty acids that are ultimately converted to ketones in the liver.

EuDKA is a result of this shift in metabolic priority. The ketosis that develops disposes patients to developing an acidosis, and this clinical scenario poses many difficulties for both patients and clinicians. If a patient develops euDKA at home, they can easily assume their diabetes is unrelated to their current symptoms due to normal fingerstick glucose readings. The lack of characteristic hyperglycemia, as is seen in DKA, makes diagnosing euDKA difficult for clinicians, as well. As a result, there is often a delay in diagnosis and treatment, which can lead to worse outcomes for patients compared to those who present with prototypical DKA. Here, we present a patient who was successfully treated for euDKA while on an SGLT-2 inhibitor, canagliflozin, after undergoing robotic sleeve gastrectomy.

## Case presentation

A 51-year-old woman with a past medical history including T2DM, hypertension, and morbid obesity underwent a laparoscopic sleeve gastrectomy with robotic assist. Prior to admission to the hospital, her home diabetes regimen consisted of canagliflozin-metformin combination pill Invokamet® and basal-bolus insulin regimen, with post-prandial NovoLog® and nighttime Soliqua®. In the three weeks prior to the surgery, her diet consisted exclusively of protein shakes three times a day with no solid foods. During this time, her Soliqua was discontinued, but NovoLog was continued at a rate consistent with the carbohydrate content of the liquid diet. Invokamet was discontinued approximately 48 hours prior to the surgery and was not resumed post-operatively. With these pre-operative dietary and medication changes, her blood sugars remained well controlled.

The morning of post-operative day (POD) 1, the patient became slightly tachycardic at 102 beats per minute. At this time, the mild tachycardia was attributed to the 5/10 pain the patient endorsed as well as to the patient not taking home medication, metoprolol, the day of the procedure. Multiple random glucose levels were checked on POD 1, the highest of which was 153 mg/dL (reference range: 70-200 mg/dL). At this time, the patient was NPO (nil per os) because an upper GI series was to be performed.

On POD 2, the patient became lethargic and exhibited continuing tachycardia. On examination, she had cool extremities and was difficult to rouse. Additional labs were performed, including an arterial blood gas (ABG), which collectively showed an arterial pH of 7.21 (reference range: 7.35-7.45), HCO_3_- of 3 mmol/L (reference range: 20-28 mmol/L), blood glucose of 160 mg/dL (reference range: 70-200 mg/dL), and anion gap (AGAP) of 37 mmol/L (reference range: 4-14 mmol/L). Her respiratory rate had increased from 18 to 30 breaths per minute and she was admitted to the ICU for worsening elevated anion gap metabolic acidosis of undetermined etiology. DKA was not initially suspected due to the repeatedly normal blood glucose levels.

Lactic acidosis was considered as a potential etiology; however, the patient was afebrile and her blood pressures were stable, making systemic hypoperfusion secondary to sepsis or blood loss unlikely. Local vascular injury as a result of operative dissection causing lactic acidosis was considered, as well; however, these etiologies were ruled out with multiple normal measured lactate levels. The upper GI series ruled out an anastomotic leak that could have explained her vital instability in the context of a potentially separate acidosis. This patient also did not have any post-operative vomiting or diarrhea, and her albumin and phosphate levels were normal. This ruled out a normal anion gap metabolic acidosis concurrent with an anion gap-raising process, such as hyperalbuminemia or hyperphosphatemia.

The patient was initially treated with two one-liter boluses of Ringer’s lactate that each contained a 100 mL solution of 8.4% sodium bicarbonate, which were given four hours apart with a plan for repeat ABG after administration. The patient responded to the sodium bicarbonate, with repeat ABG showing pH of 7.34, HCO_3_- of 7 mmol/L, and AGAP of 32 mmol/L. At this time, urinalysis showed glucosuria (>500 mg/dL) and ketonuria (80 mg/dL). Blood levels of ketones also revealed an elevated concentration at 49.8 mg/dL, and a diagnosis of euDKA was made. The patient was then put on an insulin infusion, starting at a rate of 1 unit/hour. This was titrated up to a maximum rate of 9 units/hour. During this period, the patient received careful electrolyte monitoring, including replacement of potassium, magnesium, and phosphorus, and was also receiving a 5% dextrose solution at 100 mL/hour.

With this course of treatment, the patient’s mentation and symptoms gradually improved throughout POD 3. On early POD 4, the anion gap had been closed and the insulin infusion was discontinued. The patient was encouraged to return to a normal diet and NovoLog 5 units three times a day was reintroduced and Levemir® 35 units every morning was started. The patient was ultimately transferred out of the ICU on POD 5. Further electrolyte monitoring and corrections were made that day prior to discharge on POD 6.

## Discussion

Bariatric surgeries are an increasingly common class of procedures used to treat those with morbid obesity and have been shown to significantly improve weight loss [12]. Invariably, these patients are diabetic, and DKA is a potentially life-threatening post-operative complication of bariatric surgery. One study conducted by Aminian et al. examined the rate of DKA within 90 days after bariatric surgery in a 10-year window at a single academic medical center [13]. Twelve patients were identified as having a confirmed case of DKA, of which four suffered from T2DM. However, the incidence rate of post-bariatric surgery DKA separated by diabetes subtype was 25% for T1DM and only 0.2% for T2DM - a greater than 100-fold difference. Additionally, none of the cases identified was of euDKA, highlighting the rarity of our patient’s presentation.

Given the severity and time to diagnosis of euDKA, the peri-operative management of patients on SGLT-2 inhibitors is critically important. However, there are no universal guidelines regarding the use of SLGT-2 inhibitors prior to surgery. Current recommendations by the American Association of Clinical Endocrinologists (AACE) and the American College of Endocrinology (ACE) suggest withholding SLGT-2 inhibitors 24 hours prior to elective surgery [14]. The FDA, by contrast, recommends withholding SGLT-2 inhibitors for three days prior to surgery [15]. A review published by Milder et al. recommends withholding SGLT-2 inhibitors for one to two weeks in patients undergoing bariatric surgery with significant pre-operative dietary changes, as in our patient [16]. However, these are not yet established guidelines, and other recently published case reports have also cited the need for uniform recommendations [17]. Other risk factors for developing DKA, especially in the post-operative setting, include infection, dehydration, and medication noncompliance, though these did not apply to our patient [18].

Our patient’s last dose of Invokamet was approximately 48 hours prior to her surgery, within the current recommendations outlined by the AACE and the ACE. It is likely that our patient’s pre-operative dietary changes made her more likely to develop ketosis post-operatively, however, her blood sugars were well-controlled during this period of time (blood glucose less than 130 mg/dL). Pre-operative low-calorie dietary changes are often made to reduce liver size secondary to steatohepatitis. This assists the surgeon in visualizing the relevant anatomy for the procedure, thereby reducing post-operative complications [19]. However, the benefits of this must be properly weighed against the potential increased risk of developing ketoacidosis, as suggested by Milder et al.

Thus, there may be some utility in approaching SLGT-2 inhibitor use in the pre-operative setting in a more risk-averse manner by withholding the medication for longer than what is outlined by current recommendations, provided that the blood sugars are well controlled through other means. However, more evidence is needed at this point to inform future uniform guidelines.

## Conclusions

Developing DKA in the post-operative setting after elective bariatric surgery is an uncommon complication for patients with T2DM, whereas it is relatively common for patients with T1DM. EuDKA is a rare presentation of DKA that can also occur in the post-operative setting but can be difficult to recognize clinically due to normal blood glucose levels. Clinicians should be encouraged to educate patients about the signs and symptoms of DKA when discharging post-operatively, as this can occur further after surgery than in our patient’s case, and it can occur with normal home glucose readings. More studies should be performed to determine the best practice for managing SGLT-2 inhibitor use in the pre-operative period of diabetic patients.
